# Dual Role of miR-21 in CD4+ T-Cells: Activation-Induced miR-21 Supports Survival of Memory T-Cells and Regulates CCR7 Expression in Naive T-Cells

**DOI:** 10.1371/journal.pone.0076217

**Published:** 2013-10-01

**Authors:** Katarzyna Smigielska-Czepiel, Anke van den Berg, Pytrick Jellema, Izabella Slezak-Prochazka, Henny Maat, Hilda van den Bos, Roelof Jan van der Lei, Joost Kluiver, Elisabeth Brouwer, Anne Mieke H. Boots, Bart-Jan Kroesen

**Affiliations:** 1 Department of Pathology and Medical Biology, University of Groningen, University Medical Center Groningen, Groningen, The Netherlands; 2 Department of Rheumatology and Clinical Immunology, University of Groningen, University Medical Center Groningen, Groningen, The Netherlands; 3 Department of Laboratory Medicine, University of Groningen, University Medical Center Groningen, Groningen, The Netherlands; 4 Groningen Research Initiative on healthy Ageing and Immune Longevity (GRAIL), University of Groningen, University Medical Center Groningen, Groningen, The Netherlands.; New York University, United States of America

## Abstract

Immune cell-type specific miRNA expression patterns have been described but the detailed role of single miRNAs in the function of T-cells remains largely unknown. We investigated the role of miR-21 in the function of primary human CD4+ T-cells. MiR-21 is substantially expressed in T-cells with a memory phenotype, and is robustly upregulated upon αCD3/CD28 activation of both naive and memory T-cells. By inhibiting the endogenous miR-21 function in activated naive and memory T-cells, we showed that miR-21 regulates fundamentally different aspects of T-cell biology, depending on the differentiation status of the T-cell. Stable inhibition of miR-21 function in activated memory T-cells led to growth disadvantage and apoptosis, indicating that the survival of memory T-cells depends on miR-21 function. In contrast, stable inhibition of miR-21 function in activated naive T-cells did not result in growth disadvantage, but led to a significant induction of CCR7 protein expression. Direct interaction between CCR7 and miR-21 was confirmed in a dual luciferase reporter assay. Our data provide evidence for a dual role of miR-21 in CD4+ T cells; Regulation of T-cell survival is confined to activated memory T-cells, while modulation of potential homing properties, through downregulation of CCR7 protein expression, is observed in activated naive T-cells.

## Introduction

Acquisition of effective, long-term immunity requires development of memory CD4+ T-cells. This process, induced by activation of a naive T-cell, involves extensive transcriptional activity. Consequently, naive and memory T-cells are characterized by distinct gene expression patterns [1,2]. However, not only the mere presence of transcripts, but also their regulation e.g. by microRNAs (miRNAs) is crucial for proper development and function of T-cells [3,4]. MiRNAs, a class of small, single-stranded RNA molecules, regulate gene expression at the post-transcriptional level. By binding to partly complementary target sequences in the 3’ UTR of the mRNA, miRNAs induce degradation or translational inhibition of the targeted mRNA [5]. Like coding genes, expression of miRNAs is dynamically regulated during activation and differentiation of T-cells [6-8]. Consequently, various effector T-cell subsets are characterized by distinct miRNA expression profiles [9,10]. However, the contribution of single miRNAs in the function of individual T-cell subsets is still largely unknown.

Several miRNAs are highly expressed in freshly isolated human memory CD4+ T-cells [9]. Amongst these, miR-21 has anti-apoptotic properties which have been extensively studied in pathological conditions including cancer, cardiovascular disease and autoimmunity [11-14]. Indeed, miR-21 was recently shown to suppress apoptosis and induce proliferation of primary murine and human T-cells [15-18]. In accordance with the pronounced expression in memory T-cells, it has been shown that miR-21 can be induced upon activation of CD2+ T-cells [6]. However, the kinetics and degree of miR-21 upregulation, as well as the differential functional consequences thereof in naive and memory T-cells remain unknown.

In addition to apoptosis-related genes, bioinformatic analysis [19,20] of putative miR-21 targets relevant for T-cell biology revealed several immune-related genes, including CC-chemokine receptor 7 (CCR7), which is substantially expressed on naive T-cells [21]. By binding with its ligands (CCL19 and CCL21) presented on the surface of high endothelial venules, CCR7 enables entry of T-cells into lymph nodes, and as such ensures (re)circulation of naive T-cells through the lymphatic system (reviewed in 22).

In this study we focused on the physiological role of high miR-21 expression in memory T-cells and the physiological consequences of activation-induced miR-21 expression in naive T-cells. By inhibiting endogenous miR-21 function during activation we showed that the survival of CD4+ T-cells, resulting from anti-apoptotic activity of miR-21, is confined mostly to the memory T-cell compartment. In contrast, activation-induced upregulation of miR-21 in naive T-cells post-transcriptionally regulates expression of the lymph node homing receptor CCR7. Thus, we provide evidence for a divergent role of miR-21 in two key aspects of T-cell biology, i.e. survival of memory T-cells and potential homing properties of naive T-cells. 

## Results

### MiR-21 expression is associated with memory T-cell phenotype and is induced upon αCD3/CD28 activation of CD4+ T-cells

**Figure 1:**
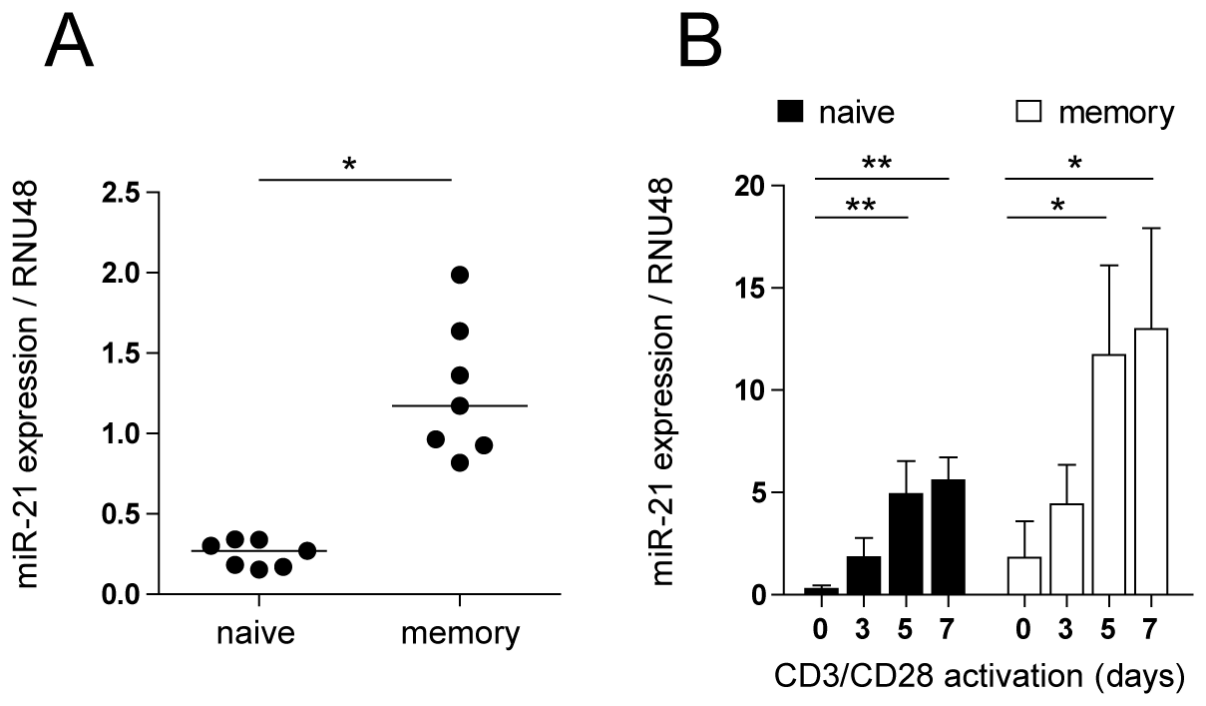
MiR-21 expression is associated with memory T-cell phenotype and is induced upon αCD3/CD28 activation of naive and memory T-cells. **A** Baseline miR-21 expression was analyzed by qRT-PCR in naive (CD4+ CD45RO-) and memory (CD4+ CD45RO+) T-cells isolated from PBMC of healthy volunteers. Relative expression normalized to the RNU48 reference gene is shown. Each dot represents a separate donor. Lines represent median values (*n=7*, Wilcoxon signed ranked test). **B** MiR-21 expression was analyzed by qRT-PCR in naive (CD4+ CD45RO-) and memory (CD4+ CD45RO+) T-cells before (day 0), and at indicated time points after activation with plate-bound-anti-CD3/soluble-anti-CD28 mAbs. Relative expression normalized to the RNU48 reference gene is shown. Data are expressed as median with interquartile range (*n=6* donors, statistical analysis inside the T-cell subset: Friedman test, with a Dunn’s Multiple Comparison test). *p<0.05, **p<0.01, ***p<0.0001.

MiR-21 expression was assessed in naive and memory CD4+ T-cells isolated from PBMC of healthy volunteers (as depicted in Figure S1A). The expression of miR-21 was detectable in both T-cell subsets, and in agreement with previous studies we observed that memory T-cells expressed higher levels of miR-21 than naive T-cells (median fold difference 5.1, p=0.016, Wilcoxon test) (Figure 1A). No differences in miR-21 expression were observed between central and effector memory T-cells, indicating that high miR-21 expression is a general feature of the memory T-cell phenotype (Figure S1B-D). We next studied the kinetics of miR-21 induction upon T-cell activation. We assessed if and to what extent αCD3/CD28 activation can induce miR-21 expression in freshly isolated naive and memory T-cells. The expression of miR-21 was strongly induced in response to activation of both naive (median fold increase 13.8 at day 7, p<0.001, Friedman test) and memory T-cells (median fold increase 4.6 at day 7, p<0.001, Friedman test), indicating that miR-21 upregulation is a general feature of T-cell activation (Figure 1B). Both T-cell subsets responded to the stimulation in a similar manner in regard to the increase in cell size (recorded by forward- and side-scatter) and expression of IL-2 receptor α (CD25). By day seven, all naive T-cells had obtained a memory phenotype as defined by acquisition of CD45RO expression (Figure S1E and F).

### MiR-21 mediates survival of activated memory CD4+ T-cells

**Figure 2:**
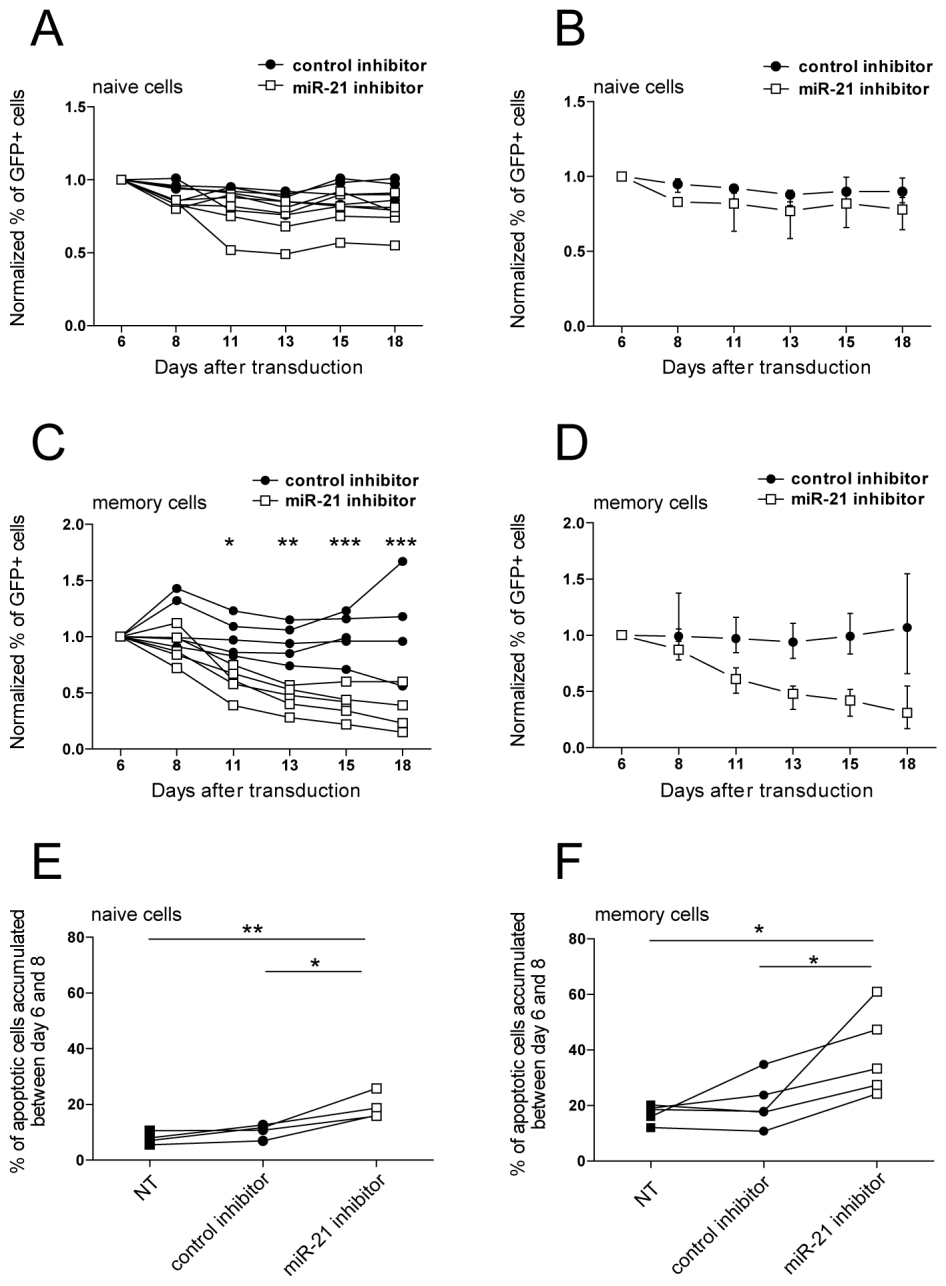
MiR-21 mediates survival of activated memory CD4+ T-cells. **A** Resting naive (CD3+ CD8-CD45RO-CD25-) T-cells were stimulated with 5µg/ml PHA and 100 U/mL IL-2, followed by transduction with lentiviral vectors harboring miR-21 or control inhibitor (scrambled hairpin sequence) and GFP. The percentage of GFP+ cells in culture over time was monitored by FACS. Data were normalized to the first measurement at day six. Each line represents a separate donor (*n*=5, two-way RM ANOVA with Bonferroni posttests, ns-not significant). B Data from A, depicted as median values with interquartile range. **C** Resting memory (CD3+ CD8-CD45RO-CD25-) T-cells were stimulated with 5µg/ml PHA and 100 U/mL IL-2, followed by transduction with lentiviral vectors harboring miR-21 or control inhibitor (scrambled hairpin sequence) and GFP. The percentage of GFP+ cells in culture over time was monitored by FACS. Data were normalized to the first measurement at day six. Each line represents a separate donor (*n*=5, two-way RM ANOVA with Bonferroni posttests). D Data from C, depicted as median with interquartile range. **E** Activated naive, and **F** activated memory GFP+ T-cells harboring miR-21 or control inhibitor were isolated by FACS from mixed cultures (A, and C respectively) at day six post lentiviral transduction. Isolated GFP+ cells, and activated not transduced cells (NT) were cultured in complete media supplemented with 100 U/mL of IL-2 for 48h (until day 8), and percentage of apoptotic cells was assessed by FACS-based measurement of mitochondrial trans-membrane potential loss, using DilC1(5). Each line represents a separate donor (naive: *n*=4, memory *n=5*, RM ANOVA with Bonferroni posttests). *p<0.05, **p<0.01, ***p<0.0001.

To determine the functional role of activation-induced miR-21 expression we stably inhibited its function using a lentiviral-based, antisense miR-21 expression system (miR-21 inhibitor, Figure S2A) in activated naive and memory T-cells. To assess if miR-21 inhibition affects the growth of activated T-cells, we performed a competition assay in which the percentage of GFP+ cells, harboring miR-21 or control inhibitor (a scrambled hairpin sequence), was monitored over 15 to 18 days in mixed GFP+ and GFP- cell cultures (as schematically depicted in Figure S2B). MiR-21 inhibition in activated naive T-cells led to a subtle, but consistent decrease in the percentage of GFP+ cells only at the beginning of the assay (day 8), and then remained stable until day 18 (Figure 2A, B). In contrast, miR-21 inhibition in activated memory T-cells led to a continuous depletion of GFP+ cells over time, indicating that survival of memory T-cells relies on the presence of miR-21 (Figure 2C, D). To assess if the observed growth disadvantage results from increased apoptosis, we FACS isolated GFP+ cells harboring miR-21 or control inhibitor at day six following transduction, and assessed apoptosis after an additional 48h culture period. Analysis of the loss of mitochondrial transmembrane potential revealed an increased apoptosis rate in both naive (Figure 2E) and memory (Figure 2F) T-cells harboring miR-21 inhibitor. The increase was most pronounced in the memory T-cells, indicating that activation-induced miR-21 expression is an important anti-apoptotic factor for T-cells in general, but is especially necessary for the maintenance of activated memory T-cells.

### MiR-21 regulates expression of CC-chemokine receptor 7 (CCR7)

**Figure 3:**
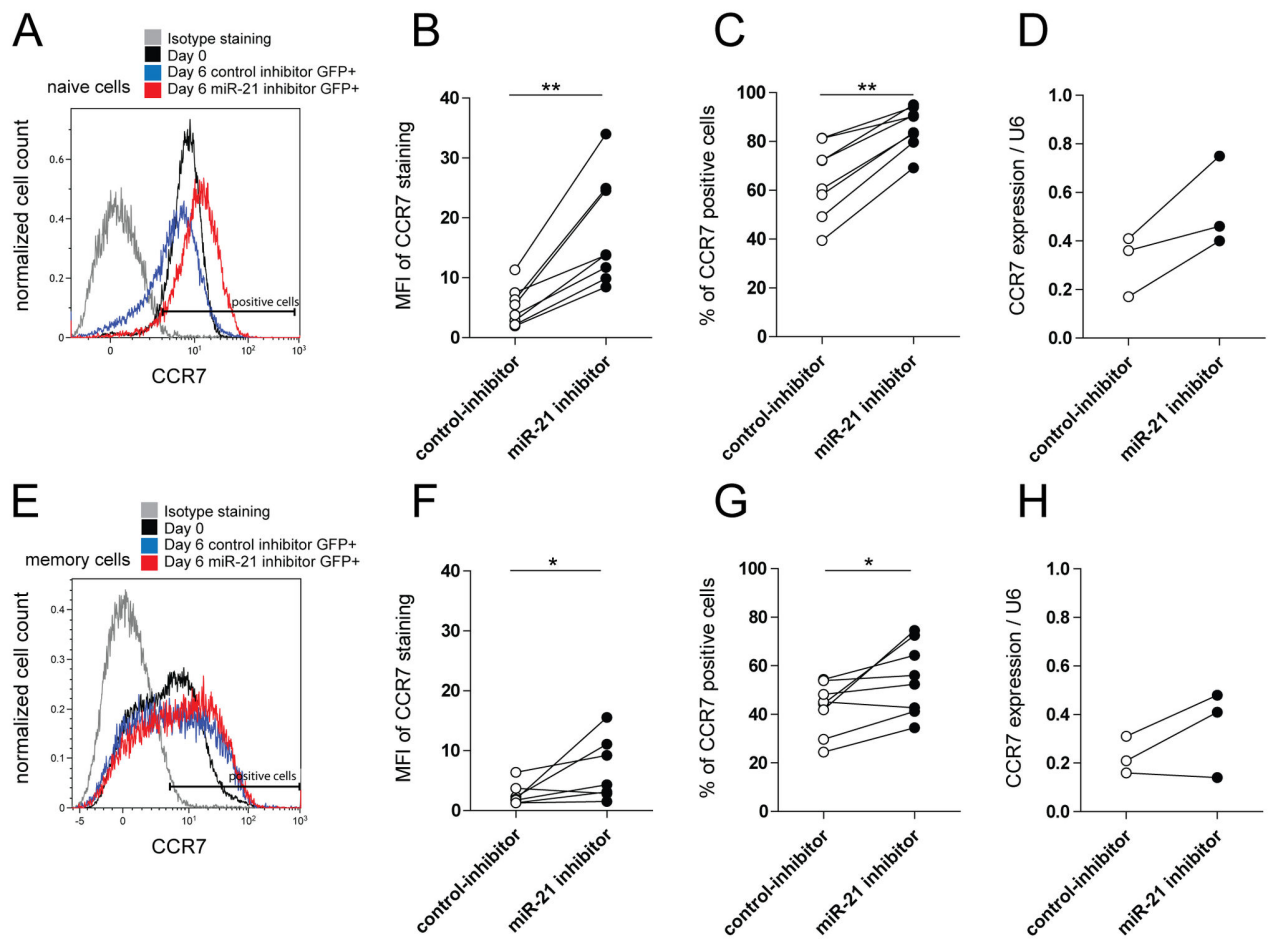
MiR-21 inhibition leads to increased CCR7 expression in activated T-cells. **A** Representative FACS staining plot depicting CCR7 expression on resting naive (CD3+ CD8-CD45RO-CD25-, Day 0), and on PHA/IL-2 activated, GFP+ cells harboring miR-21 or control inhibitor (scrambled hairpin sequence) at day six post lentiviral transduction. The isotype staining control is depicted. B Quantification of CCR7 staining of GFP+ cells from A, depicted as MFI (geometric mean fluorescent intensity). Each line represents a separate donor (*n=8*, Wilcoxon signed ranked test). **C** Percentage of CCR7 positive, GFP+ cells from A. Each line represents a separate donor (*n=8*, Wilcoxon signed ranked test). **D** GFP+, PHA/IL-2 activated naive T-cells harboring miR-21 or control inhibitor were FACS isolated at day six post-lentiviral transduction and CCR7 transcript expression was determined by qRT-PCR. Each line represents a separate donor (*n=3*, paired t-test, ns). Relative expression normalized to the U6 reference gene is shown. **E** Representative FACS staining plot depicting CCR7 expression on resting memory (CD3+ CD8-CD45RO+CD25-, Day 0), and on PHA/IL-2 activated, GFP+ cells harboring miR-21 or control inhibitor (scrambled hairpin sequence) at day six post lentiviral transduction. The isotype staining control is also depicted. F Quantification of CCR7 staining of GFP+ cells from E, depicted as MFI (geometric mean fluorescent intensity). Each line represents a separate donor (*n=7*, Wilcoxon signed ranked test). **G** Percentage of CCR7 positive, GFP+ cells from E. Each line represents a separate donor (*n=7*, Wilcoxon signed ranked test). **D** GFP+, PHA/IL-2 activated memory T-cells harboring miR-21 or control inhibitor were FACS isolated at day six post-lentiviral transduction and CCR7 transcript expression was determined by qRT-PCR. Each line represents a separate donor (*n=3*, paired- t-test, ns). Relative expression normalized to the U6 reference gene is shown. *p<0.05, **p<0.01.

**Figure 4:**
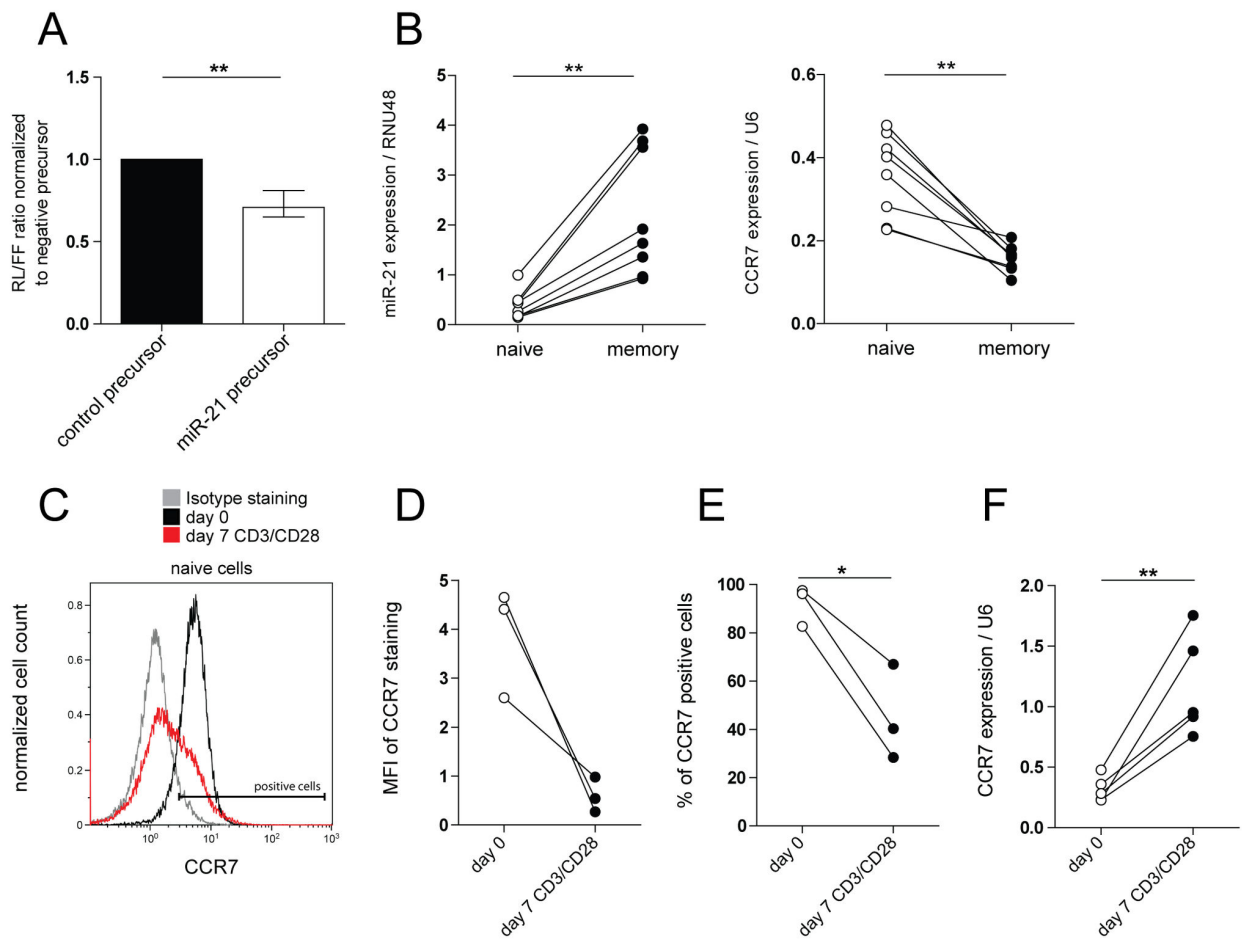
CCR7 is a direct target of miR-21 and correlates inversely with miR-21 expression in resting and activated T-cells. **A** Ratio of Renilla luciferase (RL) to firefly luciferase (FL) signal, determined in lysates of Cos-7 cells co-transfected with psiCHECK-2 construct harboring the CCR7 3’ UTR and a synthetic miR-21 or control precursor. Median values with range of data normalized to control precursor are depicted (*n=4* independent experiments, paired t-test). **B** MiR-21 and CCR7 expression levels were assessed by qRT-PCR in freshly isolated naive (CD4+ CD45RO-) and memory (CD4+ CD45RO+) T-cells. Expression levels are shown relative to the RNU48 and U6 reference genes respectively. Each line represents a separate donor (*n= 8*, Wilcoxon signed ranked test). **C** Representative FACS staining plot depicting CCR7 expression on isolated naive (CD4CD45RO-) T-cells before (day 0), and after seven days of activation with plate-bound-anti-CD3/soluble-anti-CD28 mAbs. Isotype staining is depicted. D Quantification of CCR7 staining from C, depicted as MFI (geometric mean fluorescent intensity). Each line represents a separate donor (*n=3*, paired t-test, ns). **E** Percentage of CCR7 positive, GFP+ cells from C. Each line represents a separate donor (*n=3*, paired t-test). **F** CCR7 transcript expression was determined by qRT-PCR in naive (CD4+ CD45RO-) T-cells before (day 0), and after seven days of activation with plate-bound-anti-CD3/soluble-anti-CD28 mAbs. Each line represents a separate donor (*n=5*, paired t-test). Expression levels shown in the graph are normalized to the U6 reference gene. *p<0.05, **p<0.01.

Since miR-21 appeared to function differently in naive and memory T-cells, we next set out to study the functional role of miR-21 in activated naive T-cells. To this end, we examined the effect of miR-21 inhibition on specific aspects of the transition from a naive to a memory phenotype by analyzing the expression of CD45RA, CD45RO and CCR7, defining naive, central and effector memory T-cell stages [21], as well as the expression of CD25 on resting naive, and on activated GFP+ T-cells. Changes in the expression of CD45RA, CD45RO and CD25 resulting from the activation, were similar in T-cells harboring miR-21 and control inhibitor (Figure S2C). In contrast, the expression of CCR7 was significantly increased on activated naive T-cells harboring miR-21, but not control inhibitor (Figure 3A). The increase in the expression of CCR7 was evident at the level of CCR7 expression per cell (defined as geometric mean fluorescent intensity of the staining: MFI, Figure 3B), and in the amount of CCR7 positive cells in the culture (Figure 3C). A similar result was observed at the CCR7 transcript level (Figure 3D). The expression of CCR7 protein on memory T-cells harboring miR-21 inhibitor was also increased at day six after transduction, however to a significantly lesser extent than in naive T-cells (median fold increase 2.4 and 4.1 respectively) and with more variation between different donors (Figure 3E-H). The miRNA target prediction programs TargetScan (www.targetscan.org/) [19] and MicroCosm Targets (www.ebi.ac.uk/enright-srv/microcosm/htdocs/targets/v5/) [20] denote a conserved miR-21 binding site in the 3’ UTR of the human CCR7 transcript suggesting a direct interaction between the CCR7 transcript and miR-21 (Figure S2D). Co-transfection of a dual luciferase reporter construct harboring the 3’ UTR of CCR7 together with miR-21 precursor molecules resulted in a significant decrease of the relative luciferase levels. This indicates that CCR7 is indeed a direct target of miR-21 (Figure 4A). In agreement, freshly isolated naive and memory T-cells showed inversed miR-21 and CCR7 expression patterns (Figure 4B). In addition, αCD3/CD28 activation of isolated naive T-cells led to a significant downregulation at the CCR7 protein level (Figure 4C-E), in spite of increased CCR7 transcript levels (Figure 4F), which also indicates a post-transcriptional regulation of CCR7 protein expression. Together these data show that activation-induced miR-21 expression (Figure 1B) negatively regulates CCR7 protein levels, especially in activated naive T-cells.

## Discussion

In this study we show that activation-induced miR-21 is an integral part of the T-cell activation process where it co-regulates fundamentally different aspects of T-cell biology depending on the differentiation status of the T-cell. Specifically, we show that miR-21 regulates the potential migration capacity and transition towards the memory T-cell phenotype of activated naive T-cells, and supports survival of activated memory T-cells. Likely, the different gene expression profiles characterizing naive and memory T-cells [23,24] are fundamental to the observed dual functionality of miR-21. MiRNAs can theoretically regulate a multitude of diverse target genes, however, only a fraction of the predicted target genes is co-expressed together with the miRNA in a given cell type and, as such, can be subjected to miRNA-based regulation. Moreover, the miRNA-target gene interaction is also affected by the presence of other target genes, which are competing for the same miRNA [5,25]. Thus, the overall miRNA-target gene expression ratio, and the abundance of individual target genes are likely of central importance for the factual miRNA-based regulation.

MiR-21 levels are substantially higher in *ex vivo* isolated memory T-cells than in naive T-cells (this study and [9,10]). Sub division of memory T-cells into central and effector memory phenotypes did not reveal any differences in miR-21 expression, indicating that high miR-21 levels are a general feature of the memory T-cell phenotype. It would be interesting to study miR-21 expression also in terminally differentiated (i.e. CD45RO-CCR7-) T-cells, which are virtually absent in the CD4+ population but present in the CD8+ population.

MiR-21 expression is significantly induced upon T-cell activation in both naive and memory T-cells. However, we found that memory T-cells upregulate miR-21 more rapidly and achieve higher levels of expression, indicating a more prominent role of miR-21 in this population. Expression of the primary-miR-21 transcript can be induced by mitogenic stimuli, such as phorbol-12-myristate-13-acetate (PMA) in several cancer cell lines, and is under positive transcriptional control of AP-1, STAT3 and NF-kB [26-29]. Two of these transcription factors, AP-1 and NF-κB, are directly downstream of the TcR/CD28 and PKC signaling pathways [30,31], and as such may be responsible for the observed miR-21 induction following T-cell activation. Indeed, we observed an almost complete ablation of activation-induced miR-21 expression upon PKC inhibition (data not shown).

Previous studies in mice and man have shown the involvement of miR-21 in T-cell survival in general [15,18]. Our data, discriminating between naive and memory T-cells, indicate that the requirement for miR-21-based regulation of T-cell survival is largely restricted to T-cells with a memory phenotype. Although induction of apoptosis was observed in both activated naive and memory T-cells early after miR-21 inhibition, continuous depletion of T-cells was observed only in the memory T-cell cultures. Stagakis et al. have recently reported that miR-21 affects T-cell proliferation [16]. Therefore it is possible, that the observed loss of memory T-cells lacking miR-21 is the result of enhanced apoptosis combined with a reduced proliferation rate. However, we did not observe a clear effect of miR-21 inhibition on the proliferative potential of either naive or memory T-cells (data not shown). *In vivo* studies are warranted to further study the details of the observed differential response of naive and memory T-cells towards the inhibition of miR-21 expression, especially in relation to defined aspects of long-term memory T-cell survival.

Stable inhibition of miR-21 in activated naive T-cells led to a significant upregulation of CCR7. To our knowledge this is the first report describing direct, miRNA-driven post-transcriptional regulation of CCR7 expression in human T-cells. It has been previously shown that the expression of CCR7 is downregulated following T-cell activation, and that activated T-cells within secondary lymphoid organs express lower levels of CCR7 protein [32,33]. Furthermore, decreased expression of CCR7, which is paralleled by increased expression of CXCR5, has been shown to guide activated T-cells out of the T-cell areas of the lymph node and direct them towards the B-cell follicles [34]. We found that activation-induced expression of miR-21 regulates CCR7 especially in naive T-cells. Therefore, we propose that fine-tuning of CCR7 expression by miR-21, following the activation of naive T-cells, enhances the egress of activated T-cells from the T-cell rich areas of the lymph nodes, and prevents consecutive recycling through other lymph nodes. The induction of miR-21 upon activation of naive T-cells is thus an inherent part of T-cell programming, which is actively associated with quelling of the naive T-cell state and induction of the memory T-cell phenotype. Regulation of CCR7 in activated (central) memory T-cells was also observed, however, to a much lesser extent than in naive T-cells, and with more variability between the donors. It is likely that abundant expression of other miR-21 target genes prevents regulation of CCR7 in central memory T-cells, and that the miR-21 targetome in different T-cell subsets varies based on the overall expression pool of potential miR-21 target genes. Of note, bioinformatics analysis revealed no miR-21 binding site in the 3’ UTR of the murine CCR7 transcript, denoting possible fundamental differences between the regulation of CCR7 in mice and man.

Together, our results provide evidence that miR-21 differentially affects naive and memory T-cells. We propose that intrinsic, phenotype-specific differences between these two T-cell subsets, e.g. depending on the miRNA-mRNA target gene balance, dictate the outcome of miR-21 function.

## Materials and Methods

### Ethics statement

The study was approved by The Medical Ethical Committee (METC) of the University Medical Center Groningen (UMCG) (project number: 2009.118) and written informed consent was obtained from all donors.

### PBMC isolation

Peripheral blood mononuclear cells (PBMC) from healthy donors were isolated immediately after blood withdrawal into heparin-containing vacutainer tubes (Becton Dickinson, Franklin Lakes, USA). Isolation was done by density gradient centrifugation of diluted blood (1:1 PBS) using Lymphoprep (Axis-shield, Oslo, Norway) according to the manufacturer instructions.

### Isolation and culture of CD4+ T-cell subsets

Naive (CD4+ CD45RO-) and memory (CD4+ CD45RO+) (Figure 1A, B; Figure 4B-F; Figure 1A, E, F); naive (CD4+ CD45RO-CCR7+), central memory (CD4+ CD45RO+ CCR7+) and effector memory (CD4+ CD45RO+ CCR7-) (Figure 1B, C, D); as well as resting naive (CD3+ CD8-CD45RO-CD25-) and resting memory (CD3+ CD8-CD45RO+CD25-) T-cells (Figure 2A-F; Figure 3A-H; Figure 2C) were isolated from PBMC of healthy donors by fluorescence-activated cell sorting (FACS) (MoFlo, Beckman Coulter, Brea, USA) using staining with monoclonal antibodies (mAbs) against human: CD4-FITC (Edu-2), CD8-FITC (MCD8, both from IQ Products, Groningen, The Netherlands), CD4-eFluor-450 (RPA-T4), CD45RO-PE (UCHL1), CD3-eFluor-450 (OKT-3), CD25-APC (BC96, all from eBioscience, San Diego, USA), CCR7-biotinylated (3D12, BD Biosciences) and APC-labeled streptavidin (Biolegend, San Diego, CA, USA). Cells used for assessment of baseline miR-21 expression were directly lysed with Qiazol reagent (Qiagen, Venlo, The Netherlands), and stored at -20°C until RNA extraction procedure. Isolated CD4+ T-cell subsets were cultured in RPMI 1640 with L-glutamine supplemented with 10mg/ml gentamycin and 5% Human Serum AB (all from Lonza, Basel, Switzerland) (complete media), at 37°C in 5% CO_2_. Prior to lentiviral transduction cells were stimulated for 24h in complete media supplemented with 5µg/ml phytohaemaglutinin (PHA, Thermo, Fisher Scientific Remel products, Lenexa, USA) and 100U/mL recombinant human IL-2 (Peprotech, London, England). Following lentiviral transduction cells were cultured in complete media supplemented with 100U/mL recombinant human IL-2 (Peprotech).

### Cell lines

COS-7 cells (African Green Monkey SV40-transformed kidney fibroblast cell line) and HEK293T cells (SV40 Large T-antigen-transformed human embryonic kidney cell line) were cultured in Dulbecco modified Eagle medium (DMEM) supplemented with 10% FBS, 200mM L-glutamine and 10mg/ml gentamycin (Lonza) at 37°C in 5% CO_2_. Both cell lines were obtained from ATCC.

### T-cell activation with αCD3/CD28 mAbs

TCR stimulation of isolated naive (CD4+ CD45RO-) and memory (CD4+ CD45RO+) T-cells was performed with plate-bound anti-human-CD3 and soluble anti-human-CD28 mAbs. Briefly, culture plates were incubated with goat-anti-mouse-IgG2a Ab (Cat. No. 1080-01, Southern Biotechnology) overnight at 4°C, followed by washing with PBS and 1h incubation with hybridoma-culture supernatant, containing anti-human-CD3 IgG2a mAb (clone WT32, approx. conc. 1µg IgG/mL) at RT. Unbound anti-CD3 antibody was removed by washing 4 times with excess PBS, and isolated CD4+ T-cell subsets were seeded at the density of 0.5x10^6^ cells/mL in complete medium supplemented with 5% V/V hybridoma-culture supernatant containing anti-human CD28 IgG1 mAb (clone 20-4669), giving about 0.1µg IgG/mL end concentration. Cells were re-stimulated every 2-3 days. At indicated time points, cells were harvested, stained for FACS analysis or lysed with the Qiazol reagent (Qiagen) and stored at -20°C until RNA extraction procedure.

### FACS analysis of cell surface markers

Expression of cell-surface markers on T-cell subsets before and after treatment with activating stimuli was assessed by FACS using mAbs against human CD45RO-PE (UCHL1), CD25-APC (BC96), CD45RA-eFluor605 (HI100, all from eBioscience), and CCR7-PE-Cy7 (3D12) or rat-IgG2a-PE-Cy7 isotype control (R35-9, both from BD Biosciences). To assess CCR7 expression, cells were incubated with mAb for 30min at RT. Cells were measured on BD LSR-II Flow Cytometer using Diva software (BD Biosciences) (Figure 3, Figure 2C) and on FACS Calibur flow cytometer using Cell Quest software (BD Biosciences) (Figure 4, Figure 1E, F). Data were analyzed using the Kaluza Flow Analysis Software (Beckman Coulter).

### RNA Isolation and qRT-PCR

Total cellular RNA was extracted using the miRNeasy Mini Kit (Qiagen, Venlo, The Netherlands) following the manufacturer’s instructions. The RNA quantity was measured on a NanoDrop ND-1000 Spectrophotometer (NanoDrop Technologies, Wilmington, DE). Gene expression levels were analyzed by quantitative reverse-transcription-polymerase chain reaction (qRT-PCR). For miRNA-specific cDNA synthesis, RNA was reverse transcribed using the Taqman MicroRNA Reverse Transcription kit in combination with multiplexed reverse transcription primers of TaqMan microRNA Assays (Life Technologies, Carlsbad, USA): for miR-21 (ID: 000397) and RNU48 (ID: 001006). cDNA synthesis for mRNA was performed using Superscript III RTase (Life Technologies). The qPCR reaction was performed using qPCR MasterMix Plus (Eurogentec, Liege, Belgium), and Taqman Gene expression assay for detection of CCR7: Hs01013469_m1 (Life Technologies), or gene specific primers and probe for detection of U6. Forward primer: 5’-TGGAACGATACAGAGAAGATTAGCA-3’, reverse primer: 5’-AAAATATGGAACGCTTCACGAATT-3’, and probe: 5’-6-FAM-CCCTGCGCAAGGATGACACGC-TAMRA-3’ (Integrated DNA Technologies, Coralville, USA.) RNU48 served as endogenous control for miR-21, while U6 served as endogenous control for CCR7. All reactions were run in triplicate. Mean cycle threshold (C_t_) values for all genes were quantified with the Sequence Detection Software (SDS, version 2.3, Life Technologies), using ABI7900HT thermo cycler (Life Technologies). Relative expression levels were determined using the 2^-ΔCt^ formula, where ΔCt = Ct_gene_ – Ct_ref.gene_.

### Production of viral particles, stable transduction and GFP expression assessment

Viral particles were produced by calcium phosphate (CaPO_4_)-mediated transfection of HEK293T cells with pmiRZip-21, lentiviral miR-21 inhibition vector (miR-21 inhibitor) (Cat. Nr MZIP21-PA-1), or pmiRZip-scrambled hairpin, lentiviral control inhibitor vector (control-inhibitor) (Cat. Nr MZIP000-PA-1, both from Systems Biosciences, Mountain View, USA) together with pCMV-Δ8.91 and pMD2.G expression vectors in ratio of 4:4:1. Lentiviral particles were collected 48h after transfection and passed through a 0.45µm Millex-HV filter (Millipore, Billerica, USA). Isolated, resting naive (CD3+ CD8-CD45RO-CD25-) and memory (CD3+ CD8-CD45RO+CD25-) T-cells (1x10^6^ cells/ml) were stimulated with 5µg/ml PHA (Thermo, Fisher Scientific Remel products) and 100U/mL recombinant human IL-2 (Peprotech) for 24h prior to transduction. Lentiviral transduction was carried out for 24h in the presence of 4µg/ml polybrene (Sigma-Aldrich, St. Louis, USA). After viral transduction, cells were washed three times with PBS and cultured in complete media supplemented with 100U/mL recombinant human IL-2 (Peprotech, London, England). The expression of green fluorescent protein (GFP) in mixed cultures was measured on a FACS Calibur flow cytometer (BD Biosciences), and monitored for up to 18 days. The percentage of GFP positive cells at the beginning of the assay (day 6) was set to one, and a fold difference per measurement was calculated. Transduced cells were sorted based on the GFP expression using MoFlo sorter (Beckman Coulter), or kept in mixed cultures for competition assay.

### Apoptosis measurement

Activated naive and memory GFP+ T-cells harboring miR-21 or control inhibitor were isolated by FACS from mixed cultures at day six post lentiviral transduction, and seeded in 96-wells plates in complete media supplemented with 100 U/mL of IL-2. Control, not transduced cells cultured under the same conditions were seeded alongside. Percentage of apoptotic cells was assessed after 48h (day 8) by FACS-based measurement of mitochondrial transmembrane potential loss. Briefly, cells were stained for 20min at 37°C in cell culture medium containing 50nM DilC1(5) compound (Enzo Life Sciences, NY, USA), followed by washing with PBS. Cells were kept on ice and measured directly at the FACS Calibur flow cytometer using Cell Quest software (BD Biosciences). Data were analyzed using Kaluza Flow Analysis Software (Beckman Coulter).

### Cloning of reporter construct, transient transfection and luciferase assays

The 3’ UTR sequence harboring the putative miRNA binding site and a part of the open reading frame of the human CCR7 transcript was PCR-amplified from genomic DNA using primers harboring an XhoI (5’) or NotI (3’) restriction site: 5’-TTGCTCGAGGAGACCACCACCACCTTCTC-3’ (forward) and 5’-TGGCGGCCGCCAGTGGAGCCAAGAGCTGAG-3’ (reverse), and cloned into psiCHECK2 vector (Promega, Madison, USA), as described previously [35]. The insert was sequence verified (BaseClear, Leiden, The Netherlands). Cos-7 cells (1,2x10^4^) were transfected with 125ng of the psiCHECK2 construct and 50nM Pre-miR-21 miRNA Precursor Molecule ID: PM10206 or Pre-miR miRNA Precursor Negative Control 1 (Life Technologies) using the Saint-MIX compound (Synvolux Therapeutics B.V., Groningen, The Netherlands) in 250µl serum-free medium. Four hours following the transfection, 500µl of medium supplemented with 10% FBS was added. Cells were lysed 24h after transfection and Renilla and Firefly luciferase activity was measured using the Dual-Luciferase Reporter Assay System (Promega) according to manufacturer’s instructions. For each transfection, luciferase activity was measured in duplicate with the Luminoskan Ascent Microplate Luminometer (Thermo Scientific). The Renilla over Firefly (RL/FF) luciferase ratio for miR-21 precursor was calculated. The RL/FF ratio of control precursor was set to one.

### Statistical analysis

Statistical analyses were performed in the GraphPad Prism software (version 5.0). Comparisons of paired samples were performed using the Wilcoxon signed-rank test. Comparisons of paired samples with less than six replicates were performed using the paired t-test or with repeated measures (RM) ANOVA with Bonferroni posttests. Assessment of response to the stimuli over time was performed by the Friedman test with a Dunn’s Multiple Comparison test. Comparisons of response to treatment over time between two groups were performed by the Two-way Repeated Measures ANOVA with Bonferroni posttests. All statistical analyses were two-sided, and the significance level used was *p* < 0.05. 

## Supporting Information

Figure S1 **Isolation and activation of primary human naive and memory CD4+ T-cells.** A Representative FACS plot depicting gates used for isolation of naive (CD4+ CD45RO-) and memory (CD4+ CD45RO+) T-cells. B Percentage of naive (CD45RO-/CCR7+), central memory (CD45RO+ CCR7+), effector memory (CD45RO+ CCR7-), and terminal effector (CD45RO-CCR7-) T-cells within CD4+ T-cell population, assessed by FACS, is shown. Bars represent median values with range (*n*=5 donors). C Representative FACS plot depicting gates used for purification of naive, central memory and effector memory CD4+ T-cells. D Baseline miR-21 expression analyzed by qRT-PCR in naive, central memory and effector memory CD4+ T-cells purified from PBMC of healthy volunteers. Each dot represents a separate donor. Lines represent median values (*n*=4, RM ANOVA with a Bonferroni posttests). Relative expression, normalized to the RNU48 reference gene is shown. E, F Representative FACS plots depicting phenotypic analysis of naive (CD4+ CD45RO-) E, and memory (CD4+ CD45RO+) F T-cells before (day 0) and after seven days of activation with plate-bound-anti-CD3/soluble-anti-CD28 mAbs.
(TIF)

Figure S2 **Representation of miR-21 inhibition and analysis.** **A** Schematic outline of lentiviral vector used for the inhibition of endogenous miR-21 function. Adapted from miRZips™ Lentiviral-based MicroRNA Inhibition system, Systems Biosciences. **B** Schematic representation of the GFP competition assay. **C** Representative FACS staining plots depicting expression of CD45RA, CD45RO, and CD25 on resting naive (CD3+ CD8-CD45RO-CD25-, Day 0), and on GFP+ activated T-cells, harboring miR-21 or control inhibitor, six days post lentiviral transduction. **D** Schematic indication of the miR-21 binding site in the 3’ UTR of the CCR7 gene and representation of the fragment cloned into the psiCHECK-2 dual luciferase vector.
(TIF)

## References

[1] Siegel AM, Heimall J, Freeman AF, Hsu AP, Brittain E et al. (2011) A critical role for STAT3 transcription factor signaling in the development and maintenance of human T cell memory. Immunity 35: 806-818. doi:10.1016/j.immuni.2011.09.016. 22118528 10.1016/j.immuni.2011.09.016 PMC3228524

[2] Haining WN, Ebert BL, Subrmanian A, Wherry EJ, Eichbaum Q et al. (2008) Identification of an evolutionarily conserved transcriptional signature of CD8 memory differentiation that is shared by T and B cells. J Immunol 181: 1859-1868. PubMed: 18641323. 18641323 10.4049/jimmunol.181.3.1859 PMC3771862

[3] Zhou X, Jeker LT, Fife BT, Zhu S, Anderson MS et al. (2008) Selective miRNA disruption in T reg cells leads to uncontrolled autoimmunity. J Exp Med 205: 1983-1991. doi:10.1084/jem.20080707. PubMed: 18725525. 18725525 10.1084/jem.20080707 PMC2526194

[4] Li QJ, Chau J, Ebert PJ, Sylvester G, Min H et al. (2007) miR-181a is an intrinsic modulator of T cell sensitivity and selection. Cell 129: 147-161. doi:10.1016/j.cell.2007.03.008. PubMed: 17382377. 17382377 10.1016/j.cell.2007.03.008

[5] Bartel DP (2009) MicroRNAs: Target recognition and regulatory functions. Cell 136: 215-233. doi:10.1016/j.cell.2009.01.002. PubMed: 19167326. 19167326 10.1016/j.cell.2009.01.002 PMC3794896

[6] Grigoryev YA, Kurian SM, Hart T, Nakorchevsky AA, Chen C et al. (2011) MicroRNA regulation of molecular networks mapped by global MicroRNA, mRNA, and protein expression in activated T lymphocytes. J Immunol 187: 2233-2243. doi:10.4049/jimmunol.1101233. PubMed: 21788445. 21788445 10.4049/jimmunol.1101233 PMC3159804

[7] Bronevetsky Y, Villarino AV, Eisley CJ, Barbeau R, Barczak AJ et al. (2013) T cell activation induces proteasomal degradation of argonaute and rapid remodeling of the microRNA repertoire. J Exp Med 210: 417-432. doi:10.1084/jem.20111717. PubMed: 23382546. 23382546 10.1084/jem.20111717 PMC3570096

[8] Curtale G, Citarella F, Carissimi C, Goldoni M, Carucci N et al. (2010) An emerging player in the adaptive immune response: MicroRNA-146a is a modulator of IL-2 expression and activation-induced cell death in T lymphocytes. Blood 115: 265-273. doi:10.1182/blood-2009-06-225987. PubMed: 19965651. 19965651 10.1182/blood-2009-06-225987

[9] Rossi RL, Rossetti G, Wenandy L, Curti S, Ripamonti A et al. (2011) Distinct microRNA signatures in human lymphocyte subsets and enforcement of the naive state in CD4(+) T cells by the microRNA miR-125b. Nat Immunol. 10.1038/ni.2057 21706005

[10] Wu H, Neilson JR, Kumar P, Manocha M, Shankar P et al. (2007) miRNA profiling of naive, effector and memory CD8 T cells. PLOS ONE 2: e1020. doi:10.1371/journal.pone.0001020. PubMed: 17925868. 17925868 10.1371/journal.pone.0001020 PMC2000354

[11] Chan JA, Krichevsky AM, Kosik KS (2005) MicroRNA-21 is an antiapoptotic factor in human glioblastoma cells. Cancer Res 65: 6029-6033. doi:10.1158/0008-5472.CAN-05-0137. PubMed: 16024602. 16024602 10.1158/0008-5472.CAN-05-0137

[12] Thum T, Gross C, Fiedler J, Fischer T, Kissler S et al. (2008) MicroRNA-21 contributes to myocardial disease by stimulating MAP kinase signalling in fibroblasts. Nature 456: 980-984. doi:10.1038/nature07511. PubMed: 19043405. 19043405 10.1038/nature07511

[13] van der Fits L, van Kester MS, Qin Y, Out-Luiting JJ, Smit F et al. (2011) MicroRNA-21 expression in CD4+ T cells is regulated by STAT3 and is pathologically involved in sezary syndrome. J Invest Dermatol 131: 762-768. doi:10.1038/jid.2010.349. PubMed: 21085192. 21085192 10.1038/jid.2010.349

[14] Krichevsky AM, Gabriely G (2009) miR-21: A small multi-faceted RNA. J Cell Mol Med 13: 39-53. PubMed: 19175699. 19175699 10.1111/j.1582-4934.2008.00556.x PMC3823035

[15] Meisgen F, Xu N, Wei T, Janson PC, Obad S et al. (2012) MiR-21 is up-regulated in psoriasis and suppresses T cell apoptosis. Exp Dermatol 21: 312-314. doi:10.1111/j.1600-0625.2012.01462.x. PubMed: 22417311. 22417311 10.1111/j.1600-0625.2012.01462.x

[16] Stagakis E, Bertsias G, Verginis P, Nakou M, Hatziapostolou M et al. (2011) Identification of novel microRNA signatures linked to human lupus disease activity and pathogenesis: MiR-21 regulates aberrant T cell responses through regulation of PDCD4 expression. Ann Rheum Dis 70: 1496-1506. doi:10.1136/ard.2010.139857. PubMed: 21602271. 21602271 10.1136/ard.2010.139857

[17] Iliopoulos D, Kavousanaki M, Ioannou M, Boumpas D, Verginis P (2011) The negative costimulatory molecule PD-1 modulates the balance between immunity and tolerance via miR-21. Eur J Immunol 41: 1754-1763. doi:10.1002/eji.201040646. PubMed: 21469086. 21469086 10.1002/eji.201040646

[18] Garchow BG, Bartulos Encinas O, Leung YT, Tsao PY, Eisenberg RA et al. (2011) Silencing of microRNA-21 in vivo ameliorates autoimmune splenomegaly in lupus mice. EMBO. Mol Med 3: 605-615. 10.1002/emmm.201100171 PMC3258486 21882343

[19] Garcia DM, Baek D, Shin C, Bell GW, Grimson A et al. (2011) Weak seed-pairing stability and high target-site abundance decrease the proficiency of lsy-6 and other microRNAs. Nat Struct Mol Biol 18: 1139-1146. doi:10.1038/nsmb.2115. PubMed: 21909094. 21909094 10.1038/nsmb.2115 PMC3190056

[20] Griffiths-Jones S, Saini HK, van Dongen S, Enright AJ (2008) miRBase: Tools for microRNA genomics. Nucleic Acids Res 36: D154-D158. doi:10.1093/nar/gkn221. PubMed: 17991681. 17991681 10.1093/nar/gkm952 PMC2238936

[21] Sallusto F, Lenig D, Förster R, Lipp M, Lanzavecchia A (1999) Two subsets of memory T lymphocytes with distinct homing potentials and effector functions. Nature 401: 708-712. doi:10.1038/44385. PubMed: 10537110. 10537110 10.1038/44385

[22] Förster R, Davalos-Misslitz AC, Rot A (2008) CCR7 and its ligands: Balancing immunity and tolerance. Nat Rev Immunol 8: 362-371. doi:10.1038/nri2297. PubMed: 18379575. 18379575 10.1038/nri2297

[23] Zhang W, Ferguson J, Ng SM, Hui K, Goh G et al. (2012) Effector CD4+ T cell expression signatures and immune-mediated disease associated genes. PLOS ONE 7: e38510. doi:10.1371/journal.pone.0038510. PubMed: 22715389. 22715389 10.1371/journal.pone.0038510 PMC3371029

[24] Haining WN, Angelosanto J, Brosnahan K, Ross K, Hahn C et al. (2008) High-throughput gene expression profiling of memory differentiation in primary human T cells. BMC Immunol 9: 44-2172-9-44. 18673556 10.1186/1471-2172-9-44 PMC2529265

[25] Salmena L, Poliseno L, Tay Y, Kats L, Pandolfi PP (2011) A ceRNA hypothesis: The rosetta stone of a hidden RNA language? Cell 146: 353-358. doi:10.1016/j.cell.2011.07.014. PubMed: 21802130. 21802130 10.1016/j.cell.2011.07.014 PMC3235919

[26] Löffler D, Brocke-Heidrich K, Pfeifer G, Stocsits C, Hackermüller J et al. (2007) Interleukin-6 dependent survival of multiple myeloma cells involves the Stat3-mediated induction of microRNA-21 through a highly conserved enhancer. Blood 110: 1330-1333. doi:10.1182/blood-2007-03-081133. PubMed: 17496199. 17496199 10.1182/blood-2007-03-081133

[27] Fujita S, Ito T, Mizutani T, Minoguchi S, Yamamichi N et al. (2008) miR-21 gene expression triggered by AP-1 is sustained through a double-negative feedback mechanism. J Mol Biol 378: 492-504. doi:10.1016/j.jmb.2008.03.015. PubMed: 18384814. 18384814 10.1016/j.jmb.2008.03.015

[28] Yang CH, Yue J, Fan M, Pfeffer LM (2010) IFN induces miR-21 through a signal transducer and activator of transcription 3-dependent pathway as a suppressive negative feedback on IFN-induced apoptosis. Cancer Res 70: 8108-8116. doi:10.1158/0008-5472.CAN-10-2579. PubMed: 20813833. 20813833 10.1158/0008-5472.CAN-10-2579 PMC3014825

[29] Niu J, Shi Y, Tan G, Yang CH, Fan M et al. (2012) DNA damage induces NF-kappaB-dependent microRNA-21 up-regulation and promotes breast cancer cell invasion. J Biol Chem 287: 21783-21795. doi:10.1074/jbc.M112.355495. PubMed: 22547075. 22547075 10.1074/jbc.M112.355495 PMC3381141

[30] Baier-Bitterlich G, Uberall F, Bauer B, Fresser F, Wachter H et al. (1996) Protein kinase C-theta isoenzyme selective stimulation of the transcription factor complex AP-1 in T lymphocytes. Mol Cell Biol 16: 1842-1850. 8657160 10.1128/mcb.16.4.1842 PMC231171

[31] Coudronniere N, Villalba M, Englund N, Altman A (2000) NF-kappa B activation induced by T cell receptor/CD28 costimulation is mediated by protein kinase C-theta. Proc Natl Acad Sci U S A 97: 3394-3399. doi:10.1073/pnas.97.7.3394. PubMed: 10716728. 10716728 10.1073/pnas.060028097 PMC16250

[32] Ma CS, Hodgkin PD, Tangye SG (2004) Automatic generation of lymphocyte heterogeneity: Division-dependent changes in the expression of CD27, CCR7 and CD45 by activated human naive CD4+ T cells are independently regulated. Immunol Cell Biol 82: 67-74. doi:10.1046/j.0818-9641.2003.01206.x. PubMed: 14984597. 14984597 10.1046/j.0818-9641.2003.01206.x

[33] Campbell JJ, Murphy KE, Kunkel EJ, Brightling CE, Soler D et al. (2001) CCR7 expression and memory T cell diversity in humans. J Immunol 166: 877-884. PubMed: 11145663. 11145663 10.4049/jimmunol.166.2.877

[34] Hardtke S, Ohl L, Förster R (2005) Balanced expression of CXCR5 and CCR7 on follicular T helper cells determines their transient positioning to lymph node follicles and is essential for efficient B-cell help. Blood 106: 1924-1931. doi:10.1182/blood-2004-11-4494. PubMed: 15899919. 15899919 10.1182/blood-2004-11-4494

[35] Gibcus JH, Tan LP, Harms G, Schakel RN, de Jong D et al. (2009) Hodgkin lymphoma cell lines are characterized by a specific miRNA expression profile. Neoplasia 11: 167-176. PubMed: 19177201. 19177201 10.1593/neo.08980 PMC2631141

