# Supplementary figures and images for: Dual Role of miR-21 in CD4+ T-Cells: Activation-Induced miR-21 Supports Survival of Memory T-Cells and Regulates CCR7 Expression in Naive T-Cells
Source: PLoS One. 2013 Oct 1;8(10):e76217. doi: 10.1371/journal.pone.0076217 (PMC3787993; doi:10.1371/journal.pone.0076217)

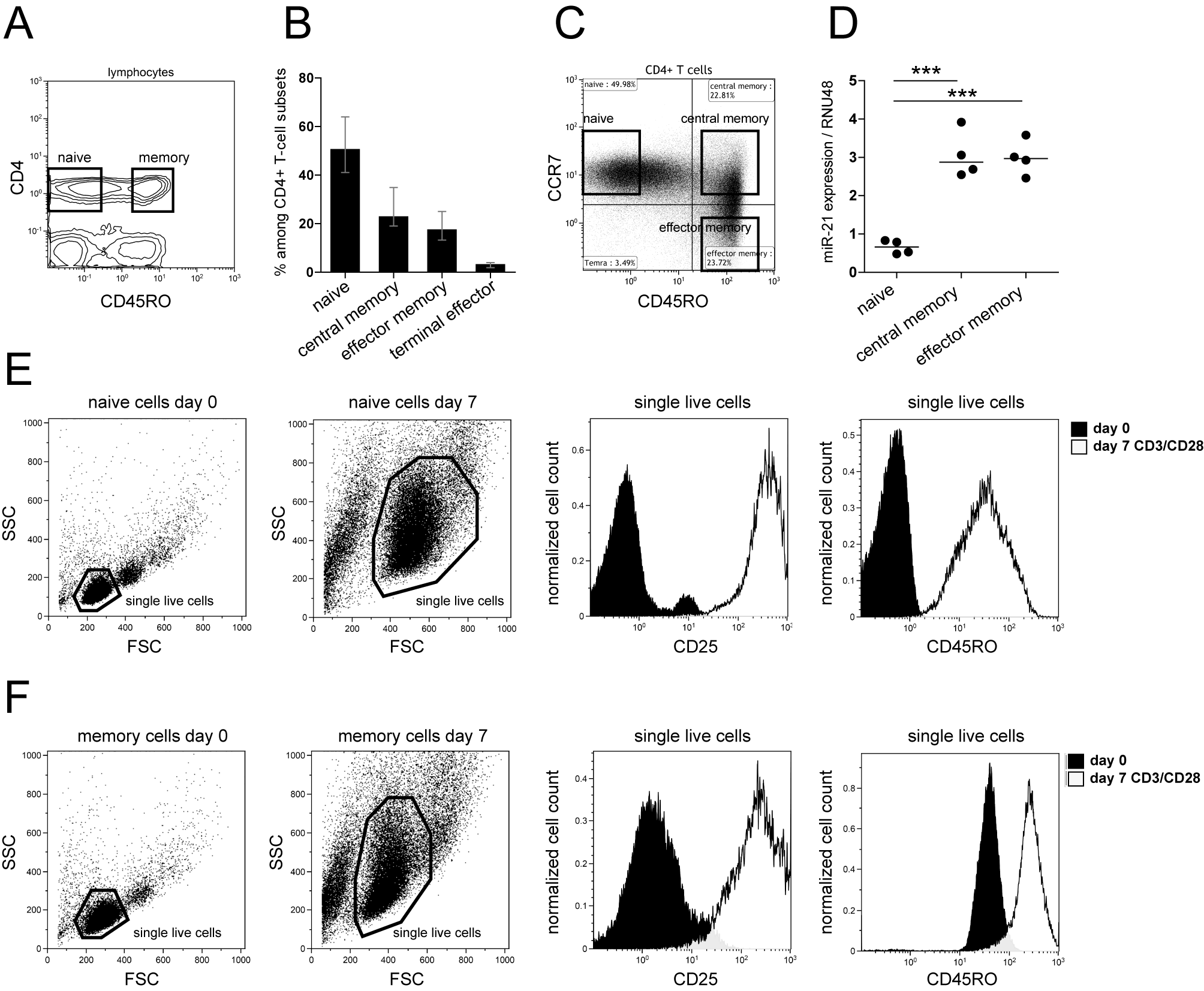

Supplement: Figure S1 — Isolation and activation of primary human naive and memory CD4+ T-cells. A Representative FACS plot depicting gates used for isolation of naive (CD4+ CD45RO-) and memory (CD4+ CD45RO+) T-cells. B Percentage of naive (CD45RO-/CCR7+), central memory (CD45RO+ CCR7+), effector memory (CD45RO+ CCR7-), and terminal effector (CD45RO-CCR7-) T-cells within CD4+ T-cell population, assessed by FACS, is shown. Bars represent median values with range (n=5 donors). C Representative FACS plot depicting gates used for purification of naive, central memory and effector memory CD4+ T-cells. D Baseline miR-21 expression analyzed by qRT-PCR in naive, central memory and effector memory CD4+ T-cells purified from PBMC of healthy volunteers. Each dot represents a separate donor. Lines represent median values (n=4, RM ANOVA with a Bonferroni posttests). Relative expression, normalized to the RNU48 reference gene is shown. E, F Representative FACS plots depicting phenotypic analysis of naive (CD4+ CD45RO-) E, and memory (CD4+ CD45RO+) F T-cells before (day 0) and after seven days of activation with plate-bound-anti-CD3/soluble-anti-CD28 mAbs. (TIF) [file pone.0076217.s001.tif]

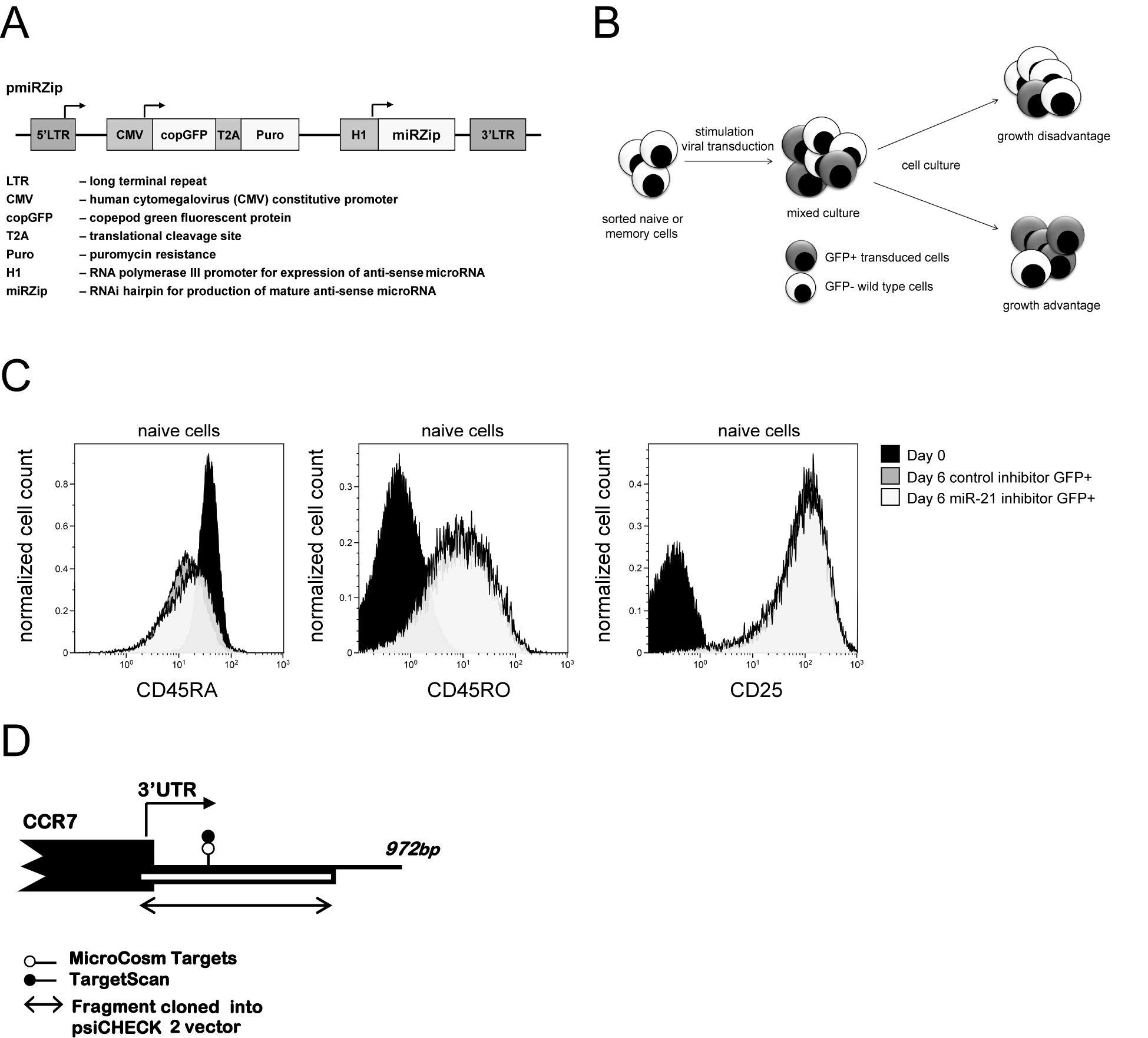

Supplement: Figure S2 — Representation of miR-21 inhibition and analysis. A Schematic outline of lentiviral vector used for the inhibition of endogenous miR-21 function. Adapted from miRZips™ Lentiviral-based MicroRNA Inhibition system, Systems Biosciences. B Schematic representation of the GFP competition assay. C Representative FACS staining plots depicting expression of CD45RA, CD45RO, and CD25 on resting naive (CD3+ CD8-CD45RO-CD25-, Day 0), and on GFP+ activated T-cells, harboring miR-21 or control inhibitor, six days post lentiviral transduction. D Schematic indication of the miR-21 binding site in the 3’ UTR of the CCR7 gene and representation of the fragment cloned into the psiCHECK-2 dual luciferase vector. (TIF) [file pone.0076217.s002.tif]
